# Establishing validity and measurement invariance of the Claremont Purpose Scale among adolescents from diverse racial–ethnic backgrounds

**DOI:** 10.1111/jora.70215

**Published:** 2026-06-08

**Authors:** Hou Xie, Kaylin Ratner, Melody Estevez, Anthony L. Burrow

**Affiliations:** ^1^ Department of Educational Psychology University of Illinois Urbana‐Champaign Champaign Illinois USA; ^2^ GripTape New York New York USA; ^3^ Department of Psychology Cornell University Ithaca New York USA

**Keywords:** adolescents, measurement invariance, race and ethnicity, The Claremont Purpose Scale, validity

## Abstract

The Claremont Purpose Scale (CPS) was designed to assess youth purpose, but the English version has not yet undergone rigorous psychometric evaluation among adolescents. Prior validation efforts have also mostly relied on white‐majority samples, raising concerns about generalizability. With 587 adolescents (M_age_ = 16.38 years, range = 13–19), confirmatory factor analyses (CFA) supported correlated three‐factor and second‐order models equivalently. However, the beyond‐the‐self dimension had the weakest connections with the overarching purpose construct and other CPS dimensions, and exploratory tests suggested that the correlated three‐factor model may have an empirical edge over the second‐order configuration. Expected zero‐order correlations provided evidence of convergent validity; up to partial scalar invariance across racial–ethnic groups was supported by multigroup CFA; and latent means testing and multigroup structural equation modeling failed to find differences between racial–ethnic groups in purpose level and adjustment associations. Findings are discussed from developmental and cultural perspectives, with implications for future adolescent purpose measurement.

Initially introduced by Frankl (1963) as a force that enables individuals to endure and overcome life's hardships, the concept of purpose has since been expanded by empirical research. This research has demonstrated that purpose not only acts as a buffer against adversity (Sumner et al., 2018), but also serves as a promoter of adaptive psychological and social outcomes, like hope and happiness (Burrow & Hill, 2011), life satisfaction and positive affect (Ratner et al., 2023), and civic engagement (Malin et al., 2015) among youth. As a result, the concept of “purpose in life” has been increasingly featured in discussions of positive youth development as a resource for growth and flourishing (Damon et al., 2003; Hill & Burrow, 2021).

Scholarship investigating purpose among youth was catalyzed by ideas put forth by Damon et al. (2003), who defined the construct with three essential components: “a *stable and generalized intention* that is at once *meaningful to the self* and at the same time leads to productive engagement with *some aspect of the world beyond the self*” (p. 121). Operationalizing these components, the Claremont Purpose Scale (CPS) was developed “for use with adolescents” (Bronk et al., 2018, p. 101). However, to our knowledge, validation efforts for the original CPS (English version) have relied exclusively on young adult samples (e.g., Anghel et al., 2021; Bronk et al., 2018; Veazey et al., 2023). This raises concerns about the scale's applicability to adolescents, particularly in light of arguments that adolescence and emerging adulthood are developmentally distinct (Arnett, 2000).

Concerns about the CPS' generalizability are not limited to the age of samples investigated. With the increasing diversity of the U.S. population, there has been a growing interest in purpose development across different cultural and racial–ethnic adolescent groups (Sumner et al., 2018). Although the CPS could potentially serve this exploratory work, such investigations have been limited given that psychometric evaluation of the English CPS has primarily been conducted in white‐majority samples. As a result, there is very little evidence that the scale performs similarly across participants from different racial–ethnic groups. Such an assumption would need to be tested to buttress more inclusive investigations of how purpose is cultivated and used as a resource for all youth in the future.

To address these gaps, this study aimed to replicate and extend the work of Bronk et al. (2018) by validating the CPS in an English‐speaking U.S. adolescent sample. We first examined how the CPS is structurally represented in adolescence and assessed convergent validity by testing its correlations with theoretically related constructs. Second, we assessed measurement invariance of the scale across racial–ethnic groups before exploring whether CPS‐based purpose levels and associations varied across groups. Together, these aims seek to enhance the utility of the CPS in adolescent research, as well as strengthen the empirical foundation for studying youth purpose development within an increasingly diverse social context.

## Measuring purpose in adolescence: The Claremont Purpose Scale

Recent academic literature has raised debates regarding the precise definition of purpose (e.g., Burrow et al., 2021; Hill & Burrow, 2021; Kashdan et al., 2024), but there is general agreement across perspectives that it serves as *a sense of intentionality*, facilitating the selection and pursuit of *personally meaningful* life aims (e.g., McKnight & Kashdan, 2009). In a landmark theoretical piece situating purpose within the purview of youth development, Damon et al. (2003) extended this understanding to include an additional component: purpose should also encompass an external component that is *beyond‐the‐self*, leading to productive engagement with a world larger than oneself. This emphasis on an external, beyond‐the‐self orientation advocated by Damon and colleagues is a hallmark of studies relying on their conceptualization of purpose.

Accordingly, researchers following this framework have put efforts into developing quantitative tools to measure youth purpose. The CPS (Bronk et al., 2018) serves as a pioneering instrument. This 12‐item self‐report scale comprises three factors—personal meaningfulness, goal directedness, and beyond‐the‐self orientation—each with four items. In the original development and validation of the CPS, Bronk et al. (2018) successfully identified the three dimensions using Exploratory Factor Analysis (EFA) and subsequently specified a second‐order structure using Confirmatory Factor Analysis (CFA), in which these subscales loaded onto a higher‐order purpose factor. This second‐order model converged with theory and demonstrated adequate psychometric properties, supporting that “the scale assesses the construct's three dimensions while also functioning as a single measure of purpose” (Bronk et al., 2018, p. 106). Moreover, CPS‐based purpose showed medium to large zero‐order correlations with conceptually related constructs, including openness, empathic concern, life satisfaction, depressive symptoms, wisdom, and purpose as measured by a widely used traditional instrument, the Purpose in Life Test (Crumbaugh & Maholick, 1964). Taken together, the CPS has emerged as a theoretically grounded measure of youth purpose. Nevertheless, as we outline below, two important issues remain unresolved: controversy regarding the CPS's underlying factor structure and lack of evidence supporting its use in adolescent populations.

### Structural considerations of the CPS

Following Bronk et al. (2018) validation, many subsequent studies examining the psychometric properties of the CPS have adopted and supported the second‐order structure (e.g., Wang et al., 2023; Wu et al., 2024). However, in a revalidation study with U.S. college students, Veazey et al. (2023) found that beyond‐the‐self items exhibited relatively low loadings and distinct patterns of association with external variables compared to the other CPS dimensions and the CPS total score. Based on these findings, the authors argued that the CPS dimensions may represent related but distinguishable facets of purpose rather than indicators of a single higher‐order construct. This interpretation is consistent with a correlated three‐factor model, which other studies have confirmed and preferred to use (e.g., Anghel et al., 2021; Noureddine et al., 2025; Tsai & Cheng, 2024). Importantly, from a statistical standpoint, correlated three‐factor and second‐order models cannot be differentiated based on model fit alone, as they are alternative rotations of the same underlying covariance structure (MacCallum et al., 1993). Consequently, differences between these representations are often interpreted considering theoretical assumptions, parameter patterns, and association with external criterion variables (e.g., Nagy et al., 2017), rather than evaluated solely based on global model fit. Thus, despite Bronk et al. (2018) openness to the flexibility of the measure, it may be wise for validation efforts to examine both configurations to clarify how the construct is represented at different hierarchical levels within a sample.

### Missing adolescent validation of the English CPS

In addition to questions concerning how the CPS dimensions are represented structurally, to our knowledge, psychometric properties of the scale's English items have not yet been evaluated in a true adolescent sample. Indeed, the initial validation study (Bronk et al., 2018) was conducted based on a sample of emerging adults aged 18–30 years, raising concerns about alignment with the scale's original intent. Arnett (2000) argued that adolescence and emerging adulthood are developmentally distinct: adolescents are usually constrained by age‐related limitations that contour their exposure to broader social, relational, and occupational domains. For adolescents, exploration in these domains is often restricted by being underage, living at home, and having limited access to spaces that facilitate ideological and interpersonal discovery (e.g., college). Identity formation is the central psychosocial task of adolescence (Erikson, 1968), but guardrails on the environment may make identity‐ and purpose‐related exploration during this period more transient and tentative than the exploration that occurs in emerging adulthood (Arnett, 2000). This is illustrated by, for example, adolescents' greater uncertainty in self‐concept and future life goals compared to emerging adults (Becht et al., 2016). Although identity development typically moves progressively toward greater levels of commitment from mid to late adolescence (Meeus et al., 2012), differences in the developmental opportunities afforded to different age groups lead us to wonder if generalizing psychometric evidence from emerging adult samples to adolescents is appropriate.

Despite these developmental constraints, adolescents can feel purposeful (Hill & Burrow, 2021) and contribute to matters beyond the self (Fuligni, 2019). Consistent with this, the CPS has been used to assess purpose in adolescent samples. For example, a study of Finnish adolescents used CPS items to assess adolescents' sense of purpose as part of a broader set of purpose‐related measures (Viljanen & Kuusisto, 2025). Similarly, Bronk et al. (2020) used the English CPS among adolescents from low‐ and middle‐income communities in the U.S., providing evidence that adolescents across different socioeconomic backgrounds reported comparable levels of purpose.

However, when it comes to examining the psychometric properties of the CPS among adolescents, more attention has been given to non‐Western cultural contexts. For example, adapted language versions of the CPS have been supported among adolescents in Mainland China (ages 11–18; Wu et al., 2024), Taiwan (ages 15–18; Tsai & Cheng, 2024), and Lebanon (ages 14–18; Noureddine et al., 2025). Across these variations of CPS, factor analyses consistently supported the presence of three distinguishable dimensions, with satisfactory model fit and strong internal reliability. The Mainland China validation further confirmed linguistic equivalence and examined differential item functioning between the Chinese and English versions, whereas the Taiwanese study provided additional evidence of the scale's test–retest reliability. However, given the significant social, cultural, and educational differences between societies, it remains unclear whether comparable psychometric properties would be observed in an American adolescent sample. Establishing whether the original English version of the CPS exhibits its expected factor structure and pattern of associations within a U.S.‐adolescent sample is therefore an important next step.

## CPS‐based purpose across diverse racial–ethnic groups

When Damon et al. (2003) first introduced their theory of youth purpose, they highlighted cultural and racial–ethnic differences in purpose as an area ripe for exploration. More than two decades have passed since the publication of this piece, and cultural diversity in the United States has significantly increased, but focus on potential variations in purpose among adolescents from diverse racial–ethnic backgrounds has not (Sumner et al., 2018). Illustrating the issue, in fall 2022, white students accounted for only 44% of public elementary and secondary school enrollment (down from 51% in fall 2012; National Center for Education Statistics, 2024). This indicates that youth of color are increasingly becoming the numerical majority in U.S. public schools. Yet, despite these trends in the demographic make‐up of America's youth, existing validation studies of the English CPS among emerging adults (Bronk et al., 2018; Veazey et al., 2023) have relied on samples that were predominantly white (~70%).

This limited representation is unfortunate given compelling evidence that purpose serves as a critical developmental asset among those from minority racial–ethnic backgrounds. Ryff et al. (2003) demonstrated that despite challenges like social inequalities and discrimination, minority status was positively associated with eudaimonic well‐being, including dimensions like life purpose and personal growth, among adolescents. Similarly, Kiang and Fuligni's (2010) research revealed that a sense of meaning and purpose in life partially mediated the link between ethnic identity and psychological adjustment outcomes among Latin, Asian, and European American adolescents. And finally, recent work during the COVID‐19 pandemic by Goodwill (2023) found that having a sense of life purpose was negatively associated with suicidal ideation, and acted as a buffer against hopelessness on suicidal thoughts, among Black adults. Importantly, these findings emerged during a period marked not only by the global public health crisis, but also by heightened racial unrest in the U.S., calling attention to the potential role of purpose as a critical psychological resource in times of profound and collective adversity. The sum of this research underscores the protective role of purpose for racial–ethnic minority groups navigating challenging circumstances.

Although this growing body of research establishes the importance of purpose for racial–ethnic minority youth, important questions remain regarding how purpose is conceptualized, structured, and experienced across racial–ethnic groups. For example, social psychological research suggests that cultural contexts and socialization can shape how individuals prioritize personal versus collective goals (Markus & Kitayama, 1991). These contextual factors may influence the way purpose is conceptualized. Researchers (e.g., Sumner et al., 2018; Wilson & Hill, 2023) have further noted that experiences of marginalization and discrimination can inspire adolescents to develop a sense of purpose that is specifically oriented toward addressing social inequities and promoting societal changes through civic or political action. Given these considerations, the relative significance of purpose dimensions, especially how individuals orient toward aims that transcend self‐interest (i.e., the beyond‐the‐self dimension), might vary across diverse populations. This could potentially affect the factor structure of existing purpose measures and impact the inferences we draw from research that employs these measures.

To examine whether the dimensions of the CPS operate equivalently across adolescents from different racial–ethnic backgrounds, measurement invariance tests can be employed. To date, measurement invariance analyses of the CPS have largely focused on gender, regions, and different age and school stages (e.g., Anghel et al., 2021; Tsai & Cheng, 2024; Wu et al., 2024; Yuliawati, 2022). The weight of this evidence suggests that the scale functions largely equivalently across groups, although some evidence points to partial scalar invariance for the beyond‐the‐self dimension across adolescence stages and school regions (Wu et al., 2024).

Although these existing measurement invariance findings are promising, it remains unclear whether comparable invariance holds across other demographic characteristics, like racial–ethnic identification. This issue is of increasing importance given the growing ethnic and racial diversity of the U.S. adolescent population. If measurement invariance is established, it provides a foundation for interpreting observed group differences beyond measurement‐related artifacts (Millsap, 2011). Such differences, if present, may reflect variations in sociocultural experiences and opportunity structures that shape how purpose is expressed (Burrow et al., 2021; Sumner et al., 2018). Invariance would also permit more defensible examinations of how CPS‐based purpose relates to other psychosocial constructs across racial–ethnic groups. If associations between purpose and psychosocial outcomes differ across groups, this would suggest that, as a developmental asset and resource, purpose cannot be assumed to function uniformly.

## The present study

With the growing emphasis on purpose in positive youth development, there is a clear need for a valid scale to measure purpose among adolescents. As such, the first aim of this study was to conceptually replicate and extend Bronk et al. (2018) validation of the CPS by verifying its factor structure in a sample of middle‐to‐late adolescents. Informed by the theoretical framework of the CPS and ongoing discussions regarding its structural representation, we first hypothesized that both a correlated three‐factor model and a second‐order model would demonstrate adequate model fit, given that they imply an equivalent underlying item covariance structure. Second, to establish convergent validity, we hypothesized that the CPS total score and its three subscales would be positively associated with each other, life satisfaction, and openness, and negatively associated with depressive symptoms. Following Bronk et al. (2018) initial validation work on the CPS, these variables were selected based on the theoretical perspectives that position purpose as an indicator of psychological well‐being (Ryff & Keyes, 1995), as well as a dispositional orientation toward curiosity and exploratory engagement (Silvia & Kashdan, 2021). In addition, we expected positive associations between CPS‐based purpose and purpose as assessed by the Life Engagement Test (LET; Scheier et al., 2006). This choice allowed us to help situate the CPS within a broader network of established purpose measures.

In our second aim, we explored whether the CPS operates comparably across adolescents from different racial–ethnic groups. We hypothesized that the overall structure of the CPS would be largely consistent across groups; however, considering potential cultural and racial–ethnic variation in how purpose is conceptualized, we remained open to identifying differences in how specific dimensions of CPS‐based purpose are represented across groups. We were particularly interested in exploring how the beyond‐the‐self dimension and its items are situated within the broader construct of purpose across adolescents from diverse racial–ethnic groups.

However, if metric and scalar measurement invariance could be successfully established, providing support for the valid use of CPS across racial–ethnic groups, we intended to explore two additional questions. First, we examined whether CPS‐based purpose differed across racial–ethnic groups at both the total and subscale levels. Second, we tested whether racial–ethnic group membership moderated the associations between CPS‐based purpose and key adjustment outcomes. These exploratory analyses would enhance our understanding of how sociocultural backgrounds influence the formation and function of purpose among youth from diverse backgrounds. Together, this work provides an essential foundation for measuring and supporting youth purpose development across groups, directly responding to calls for greater cultural responsiveness and inclusivity in psychological measurement (see Diemer et al., 2015).

## METHOD

### Participants and procedure

The sample for this study began with 587 adolescents preparing for a self‐driven learning program called *GripTape* (https://www.griptape.org). GripTape is a U.S.‐based youth program committed to providing out‐of‐school learning opportunities for young people from under‐resourced backgrounds. Over a 10‐week period, participants were encouraged to choose a topic of interest that is typically not offered in traditional school curricula (e.g., music production, business management, computer programming). Each participant was paired with an affiliated (but nonexpert) adult for emotional support and consistency throughout the program, as well as a modest stipend ($500) to facilitate their learning. By enabling adolescents to pursue personally meaningful topics, this program has been argued to represent a learning context that is particularly conducive to purpose development (Ratner et al., 2025).

Consistent with GripTape's mission to support adolescents from different backgrounds, the sample was highly diverse in terms of racial–ethnic composition (Asian‐American/Asian [30.32%], African‐American/Black [20.44%], Hispanic/Latinx [16.18%], white/Caucasian [19.25%], Native American [0.34%], Native Hawaiian or Other Pacific Islander [0.17%], multiracial [11.6%], or an unlisted racial–ethnic category [1.36%]). Females represented the majority (66.4%) of participants, whereas males made up 27.4%. A small percentage (5.3%) identified as gender minorities, including transgender, nonconforming, or other gender identities not listed. In terms of age, the analytic sample had a mean age of 16.38 years (SD = 1.24, range = 13–19). Notably, only one participant was 13 years old and sixteen were 19 years old, indicating that most of the sample fell within middle to late adolescence.

For the first aim, all 587 participants were included in the analysis to examine the factor structure and convergent validity of the CPS. For the second aim of measurement invariance testing, the analysis was constrained to the four racial–ethnic groups having the most substantial representation within the sample, namely, African‐American/Black (*n* = 120), Asian‐American/Asian (*n* = 178), Hispanic/Latinx (*n* = 95), and white/Caucasian (*n* = 113) groups. This subset comprised 506 adolescents.

Before initiating GripTape, participants completed a 30‐min online baseline survey including all variables relevant to the current study. Consent was obtained from all participants or their guardians (if participants were under 18) prior to survey administration, and they received $5 USD compensation upon completion. These procedures were approved by the Human Subjects Institutional Review Board at Cornell University.

### Measures

#### Claremont purpose scale

The CPS is a 12‐item self‐report measure designed to assess youth purpose across three dimensions (Bronk et al., 2018). Each dimension is represented by a subscale containing four items: personal meaningfulness (e.g., “How well do you understand what gives your life meaning?”), goal directedness (e.g., “How hard are you working to make your long‐term aims a reality?”) and beyond‐the‐self orientation (e.g., “How often do you find yourself hoping that you will make a meaningful contribution to the broader world?”). In this study, participants rated each item on a four‐point Likert scale, with higher scores indicating greater levels of purpose. The internal reliabilities for the overall scale (*α* = .85) and each dimension (personal meaningfulness [*α* = .89], goal directedness [*α* = .81], beyond‐the‐self orientation [*α* = .85]) were strong.

#### Life satisfaction

Life satisfaction, defined as a global assessment of a person's life quality based on their own chosen criteria, was measured using the Satisfaction with Life Scale (SWLS; Diener et al., 1985). This is a unidimensional five‐item self‐report scale, rated on a five‐point Likert scale. Sample items included “In most ways my life is close to my ideal” and “So far I have gotten the important things I want in life.” Participants rated the items based on the extent to which they agreed with each statement, and responses were averaged to generate a total life satisfaction score. The internal reliability of the scale was excellent (*α* = .85).

#### Depression

Depression was assessed using the Center for Epidemiological Studies Depression Scale for Children (CES‐DC; Faulstich et al., 1986). This 20‐item scale asked participants to indicate how much they have felt or behaved in ways described by each item over the past week. Example items included “I felt like crying” and “It was hard to get started doing things.” Each item was rated on a four‐point Likert scale. The total score was computed by averaging responses, with higher scores indicating greater depressive symptoms. Internal consistency of the scale in this study was high (*α* = .93).

#### Openness

Openness, one of the Big Five personality traits, was measured using the short form of the International Personality Item Pool‐Five‐Factor Model (Mini‐IPIP; Donnellan et al., 2006). The Mini‐IPIP consists of 20 items, with four items assigned to each Big Five dimension. Openness captures individual differences in the tendency to be receptiveness to new aesthetic and intellectual experiences (McCrae & Costa Jr., 1997). Participants rated items associated with openness (e.g., “have a vivid imagination”) on a five‐point Likert scale to indicate how accurately the statements described their experiences. Their responses were averaged to form a composite score for the openness characteristic. The internal consistency of this scale (*α* = .66) was acceptable considering the brevity of the scale (see Xiao et al., 2024).

#### The Life Engagement Test

The Life Engagement Test (LET) is a six‐item scale designed to measure purpose in life, defined as the extent to which individuals engage in activities that reflect their personal values (Scheier et al., 2006). It has been widely adopted in the literature as a measure of purpose in adolescent populations (e.g., Barcaccia et al., 2023; Kuhlman et al., 2021; Pfund et al., 2020). Unlike the CPS, this scale is unidimensional, with all items loading onto a single factor. Sample items include “To me, the things I do are all worthwhile” and “I don't care very much about the things I do” (reverse‐scored). Participants rated the items on a five‐point Likert scale, with higher scores indicating greater agreement with each item and a stronger sense of purpose. Responses were averaged to generate a total LET purpose score, which demonstrated good internal consistency (*α* = .82).

### Analytic strategy

#### Factor structure and convergent validity of the CPS

Data preparation and analysis were performed in R (Version 4.3.1). The factor structure of the CPS among our adolescent sample was assessed through confirmatory factor analysis (CFA) with the *lavaan* package (Rosseel, 2012). Two CFA models were specified: a correlated three‐factor model with four items loading on each factor, and a second‐order model with three first‐order factors loading onto a single higher‐order factor. Following the criteria established by Hu and Bentler (1999), good model fit was determined by comparative fit index (CFI) and Tucker‐Lewis index (TLI) values ≥ 0.95, a root mean square error of approximation (RMSEA) value ≤ 0.06, and a standardized root mean square residual (SRMR) value ≤ 0.08. The strength of factor loadings was evaluated using Comrey and Lee's (1992) thresholds: 0.32 (poor), 0.45 (fair), 0.55 (good), 0.63 (very good), and 0.71 (excellent). Next, convergent validity was examined by assessing zero‐order bivariate correlations between the CPS total score and subscale scores and theoretically related constructs: life satisfaction, depressive symptoms, openness, and LET‐based purpose.

#### Racial–ethnic measurement invariance of the CPS

After confirming the factor structure and convergent validity of the CPS, all subsequent analyses were conducted in parallel under both the correlated three‐factor model and the second‐order model. We presented both sets of analyses to support flexible use of the scale in ways that best serve the research goals.

The main intent of the second aim was to investigate the measurement invariance (MI) of the CPS across racial–ethnic groups. For each structure, multigroup CFA was conducted following a hierarchical framework (Chen et al., 2005; Horn & McArdle, 1992) testing configural, metric, and scalar invariance through a sequence of nested models. For the correlated three‐factor model, configural invariance was first evaluated by specifying the same three‐factor structure across groups with all parameters freely estimated. This test assessed whether the same general structure held across groups. Metric invariance was then tested by constraining item loadings onto their respective latent factors to equality across groups. This test indicated whether the items showed comparable relations to the latent factors. Scalar invariance was subsequently assessed by adding constrained intercepts of the observed indicator variables. This test provided the level of invariance typically needed to support comparisons of latent means across groups. After the standard measurement invariance sequence, we conducted an additional model‐specific invariance test to evaluate whether relations among the three latent dimensions were comparable across racial–ethnic groups. This was accomplished by constraining factor covariances to be equal across groups and checking model fit against that of a model containing unconstrained factor covariances.

For the second‐order model, additional constraints were imposed to reflect its higher‐order structure (Chen et al., 2005). Configural invariance specified the same second‐order factor structure across groups with freely estimated parameters. Metric invariance was tested in two stages: first by constraining first‐order factor loadings (items onto first‐order factors), and then by additionally constraining second‐order loadings (first‐order factors onto the higher‐order purpose factor). Following metric invariance, scalar invariance was evaluated by a similar two‐step procedure: constraining the intercepts of observed indicators (item intercepts), followed by constraining the intercepts of first‐order latent factors. When testing first‐order factor intercept invariance, the marker‐indicator method was used for model identification, with the intercept of one item per first‐order factor fixed to zero (Chen et al., 2005; Rudnev et al., 2018). This hierarchical testing approach, moving from less to more restrictive models, has been argued to imply increasingly robust evidence that the measurement instrument functions consistently across different groups (Horn & McArdle, 1992).

In the MI procedure, the fit of each model was evaluated and compared against the cutoffs established by Hu and Bentler (1999). In conjunction with the absolute value model fit indices, adjacent models were compared with likelihood ratio tests (LRTs). If adding more constraints on parameters does not substantially alter the model fit, it tends to indicate that a higher level of invariance had been achieved. A nonsignificant change in *χ* ^2^ across nested models is one conventional indicator of invariance. However, the *χ* ^2^ statistic is sensitive to sample size, inflating Type I error rates in large samples (Cheung & Rensvold, 2002; Putnick & Bornstein, 2016). This may be particularly problematic for multigroup analyses with unequal group sizes. Although group size imbalance in the present study did not reach levels previously noted as concerning (Yoon & Lai, 2018), we also used changes in alternative fit indices, including the CFI, RMSEA, and McDonald's noncentrality index (MNCI), to assess measurement invariance. In line with Meade et al. (2008) suggestions, ΔCFI < .002, ΔMNCI < .008, and ΔRMSEA < .007 were used as values that signaled invariance. Complementarily, a more sensitive index recommended in the recent measurement invariance literature, the RMSEA associated with the chi‐square difference test (RMSEA_D_), was also computed (Savalei et al., 2024). For RMSEA_D_, larger values reflect greater deterioration in model fit. Consistent with prior work (Zhang et al., 2025), RMSEA_D_ values above .08 tend to indicate substantial misfit.

Finally, when full invariance could not be established, partial invariance was tested by sequentially freeing parameters with the largest modification indices while retaining constraints on the remaining parameters (Byrne et al., 1989). Partial invariance was deemed acceptable and considered sufficient for substantive group comparisons when less than 20% of the tested loadings or intercepts were freely estimated (Dimitrov, 2010) and more than half of the items per factor remained invariant (Steenkamp & Baumgartner, 1998).

#### Potential racial–ethnic group differences in purpose and moderating effects

Conditional on the establishment of at least partial scalar invariance, latent mean comparisons of CPS‐based purpose were conducted at both the global and dimensional levels. We compared the unconstrained model, in which latent means were freely estimated across racial–ethnic groups, with a nested model in which the relevant latent means were constrained to be equal (Chen et al., 2005). A significant deterioration in model fit for the equality‐constrained model was interpreted as evidence of latent mean differences. Additionally, to examine whether racial–ethnic group membership moderated associations between CPS‐based purpose and adjustment outcomes, we estimated multigroup structural equation models (SEMs) under both factor structures. Each model incorporated the established CPS measurement model, with depression, life satisfaction, and openness specified as outcomes. For each factor structure, moderation was tested by constraining the regression paths between CPS‐based purpose and the adjustment outcomes to equality across groups and evaluating whether model fit worsened. Gender and age were included as covariates.

### Estimation strategy

Although categorical least squares estimators (e.g., WLSMV) are recommended for ordinal indicators with fewer than five response categories (Rhemtulla et al., 2012), their application is highly sensitive to asymmetric category thresholds and zero cell frequencies. In the present data, preliminary data screening suggested that several racial–ethnic groups exhibited empty response categories on some items (items 6, 7, and 12), making categorical estimation infeasible in the multigroup context. Accordingly, all analyses were conducted using robust maximum likelihood (MLR) to mitigate issues associated with sparse categorical distributions. Importantly, in recent methodological literature, treating ordinal items with three to six response categories as continuous in a linear factor model has been considered a defensible approach, particularly when robust inference is employed (Robitzsch, 2020).

## RESULTS

### Factor structure and convergent validity of the CPS

**FIGURE 1:**
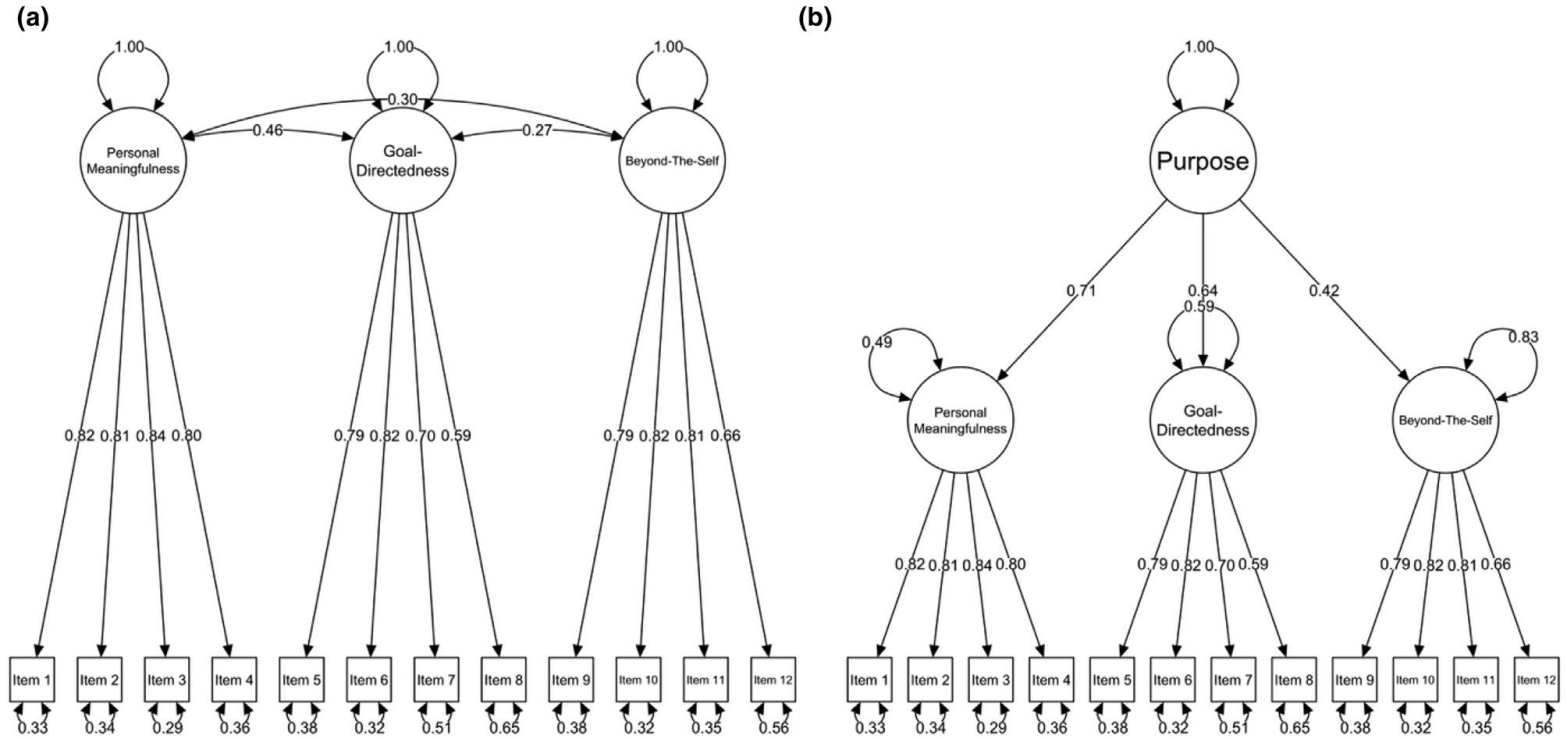
The correlated three‐factor and second‐order CPS models.

Consistent with our planned testing, two CPS models were specified in the CFA: a three‐factor model with correlated factors (Figure 1A) and a second‐order model (Figure 1B). Both demonstrated identically adequate fit and met recommended fit criteria (*χ* ^2^[51] = 111.70, *p* < .001; CFI = .98; TLI = .97; SRMR = .05; RMSEA = .05). Standardized loadings for the four personal meaningfulness items ranged from .80 to .85, those for goal directedness ranged from .59 to .82, and those for beyond‐the‐self orientation ranged from .67 to .79. These results indicated that individual CPS items are generally good representations of their corresponding factor assignments.

The two models differed only in how associations among the three dimensions were parameterized. In the correlated three‐factor model, inter‐factor correlations were small to moderate. The strongest association among the factors was between personal meaningfulness and goal directedness (*r* = .46). Associations between factors involving beyond‐the‐self orientation were relatively weaker (*r*s = .27 & .30). In the second‐order model, the loadings of the three dimensions onto the overarching purpose factor were .71 for personal meaningfulness, .64 for goal directedness, and .42 for beyond‐the‐self orientation. Again, beyond‐the‐self orientation showed the weakest association with the higher‐order purpose construct, as reflected by its acceptable, but less‐than‐ideal, second‐order loading based on Comrey and Lee's (1992) guidelines.

In an exploratory step,^1^ we compared the two structural models illustrated in Figure 1A,B by introducing external criterion variables into both models (Nagy et al., 2017). This procedure resulted in two extended models that differed in degrees of freedom, making it possible to test which model provided a more useful representation of the CPS factor structure. In these extended models (see Figure S1A,B), the criterion variables were permitted to covary with one another and with the relevant CPS latent factor structure: the three first‐order CPS factors in the correlated three‐factor model, and the higher‐order purpose factor in the second‐order model. Using the *net()* function in the *semTools* package (Jorgensen et al., 2026), we confirmed that the two extended models were indeed nested and therefore directly comparable. Both extended models demonstrated good fit, but the extended correlated three‐factor model fit the data significantly better than the extended second‐order model, Δ*χ* ^2^ (8) = 42.70, *p* < .001.

After examining the factor structure of the CPS, we evaluated its convergent validity by examining its unadjusted bivariate associations with theoretically relevant constructs. Descriptive statistics and zero‐order correlations among all study variables are presented in Table 1. As expected, the CPS total and subscale scores were positively associated with LET‐based purpose, life satisfaction, and openness, and negatively associated with depressive symptoms.

**TABLE 1:** Descriptive statistics and zero‐order correlations.

Variable	*M*	SD	1	2	3	4	5	6	7
1. CPS‐Total	3.27	0.49	—						
2. CPS‐Personal Meaningfulness	2.91	0.80	.83**	—					
3. CPS‐Goal Directedness	3.33	0.59	.74**	.43**	—				
4. CPS‐Beyond‐The‐Self	3.57	0.57	.64**	.26**	.25**	—			
5. Openness	3.97	0.65	.22**	.11*	.15**	.24**	—		
6. Depression	1.16	0.65	−.38**	−.46**	−.21**	−.12*	−.06	—	
7. Life Satisfaction	3.12	0.89	.46**	.56**	.27**	.12*	−.02	−.51**	—
8. LET‐based Purpose	3.97	0.67	.65**	.66**	.44**	.27**	.19**	−.51**	.52**

*Note*: *p ≤ .01, **p ≤ .001.

### Racial–ethnic measurement invariance of the CPS

A series of MI tests evaluated whether the CFA models presented in Figure 1A,B demonstrated equivalent psychometric properties across African‐American/Black, Asian‐American/Asian, Hispanic/Latinx, and white/Caucasian adolescents (see Table 2). Under the correlated three‐factor structure, Model 1 (configural invariance) demonstrated a good fit to the data. This suggests the factor structure was appropriate for all four groups. To evaluate metric invariance, items loadings onto their respective latent factors were constrained to equality in Model 2. Although ΔCFI (−.003) and ΔMNCI (−.013) exceeded the recommended cutoffs, most fit indices (△*χ* ^2^ [27] = 35.31, *p* = .054; ΔRMSEA = .001; RMSEA_D_ = .025) did not indicate substantial deterioration in model fit. Because the overall pattern of fit indices failed to reject the null hypothesis, we retained the constrained model and interpreted the results as consistent with metric invariance. Then, in Model 3, scalar invariance was tested by constraining item intercepts across groups. The *χ* ^2^ difference test was statistically significant (Δ*χ* ^2^ [36] = 54.56, *p* < .05), indicating a substantial deterioration in model fit when groups were assumed to have equal item intercepts. Additionally, both ΔCFI (−.007) and ΔMNCI (−.018) exceeded recommended thresholds for establishing measurement invariance. Collectively, most fit indices provided evidence that full scalar invariance could not be supported. Modification indices identified noninvariant intercepts for Items 3 and 10. After freeing these intercepts (Model 3a), changes in fit indices were negligible relative to Model 2, supporting partial scalar invariance. Lastly, after this standard measurement invariance procedure, constraining the covariances among the three latent factors to equality across groups (Model 4) did not result in a significant decrement in model fit. This finding suggested that relations among the three dimensions were comparable across racial–ethnic groups.

**TABLE 2:** Model fit indices and changes in fit for measurement invariance models.

	*χ* ^2^(df)	CFI	TLI	RMSEA	MNCI	△*χ* ^2^ (△df)	△CFI	△MNCI	△RMSEA	RMSEA_D_
Correlated three‐factor model invariance										
Mode 1 Configural invariance	251.41 (204)	0.98	0.98	0.05	0.94					
Model 2 Metric invariance	286.89 (231)	0.98	0.98	0.05	0.92					
Model 3 Scalar invariance	340.78 (267)	0.97	0.97	0.05	0.90					
Model 3a Partial scalar invariance^a^	313.25 (261)	0.98	0.98	0.04	0.93					
Model 4 Partial scalar and covariance invariance^a^	319.56 (270)	0.98	0.98	0.04	0.93					
Model 1 – Model 2						35.31 (27)	−0.003	−0.013	0.001	0.025
Model 2 – Model 3						54.56* (36)	−0.007	−0.018	0.003	0.032
Model 2 – Model 3a						25.69 (30)	0.001	0.003	−0.004	0.000
Model 3a – Model 4						7.14 (9)	0.001	0.000	−0.004	0.000
Second‐order model invariance										
Model 5 Configural invariance	251.41 (204)	0.98	0.98	0.05	0.94					
Model 6 First‐order metric invariance	286.89 (231)	0.98	0.98	0.05	0.92					
Model 7 First‐ and second‐order metric invariance	290.28 (237)	0.98	0.98	0.05	0.92					
Model 8 Item scalar invariance	333.19 (264)	0.97	0.97	0.05	0.91					
Model 8a Item partial scalar invariance^b^	308.88 (255)	0.98	0.98	0.04	0.92					
Model 9 Item partial and first‐order scalar invariance^b^	315.18 (264)	0.98	0.98	0.04	0.92					
Model 5 – Model 6						35.31 (27)	−0.003	−0.013	0.001	0.025
Model 6 – Model 7						4.42 (6)	0.000	−0.001	−0.001	0.000
Model 7 – Model 8						44.02* (27)	−0.006	−0.015	0.003	0.036
Model 7 – Model 8a						18.32 (18)	0.000	0.000	−0.002	0.006
Model 8a – Model 9						6.13 (9)	0.001	0.002	−0.002	0.000

*Note*: *p ΔCIF, ΔMNCI, and ΔRMSEA were computed by subtracting the fit indices of the less constrained model from those of the more constrained model. Models 1 and 5, as well as Models 2 and 6, are mathematically equivalent parameterizations and therefore yield identical fit indices.

^a^ Item 3 and 10 intercepts freely estimated.

^b^ Item 3, 10 and 12 intercepts freely estimated.

Under the second‐order structure, Model 5 demonstrated a good fit to the data, indicating that configural invariance was supported across racial–ethnic groups. Constraining the first‐order factor loadings (Model 6) yielded results identical to those obtained under the correlated three‐factor model (Model 2), as these constraints involve the same item‐to‐factor loadings. Thus, first‐order metric invariance across racial–ethnic groups was supported. Next, equality constraints were imposed on the second‐order loadings (Model 7), such that the three first‐order factors were constrained to load equally onto the overarching purpose factor across groups. Changes in model fit were negligible, providing support for second‐order metric invariance across racial–ethnic groups. For full scalar invariance (Model 8), constraining item intercepts across groups resulted in a significant deterioration in model fit (Δ*χ* ^2^ [27] = 44.02, *p* < .05), with both ΔCFI (−.006) and ΔMNCI (−.015) exceeding recommended thresholds. This indicated that full scalar invariance at the item level was not supported. Modification indices identified noninvariant intercepts for Items 3, 10, and 12. After freeing these intercepts (Model 8a), fit changes were minimal relative to the metric model (Model 7), supporting partial scalar invariance. Finally, at the higher‐order stage (Model 9), equality constraints on the first‐order factor intercepts did not result in substantial deterioration in model fit, supporting higher‐order scalar invariance.

**FIGURE 2:**
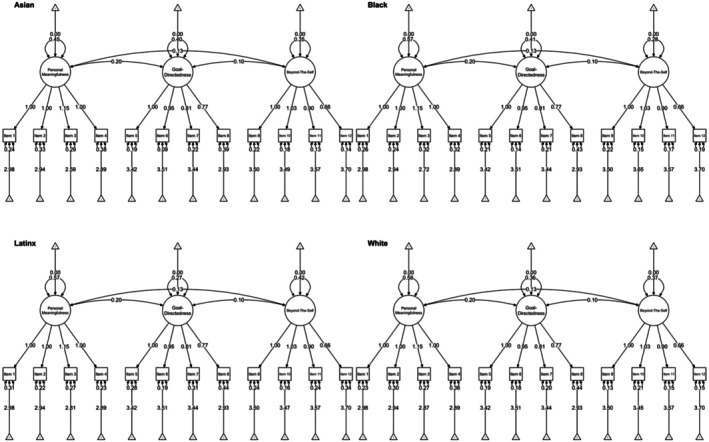
Correlated three‐factor model with partial scalar and inter‐factor invariance (Model 4). Item 3 and 10 intercepts freely estimated.

**FIGURE 3:**
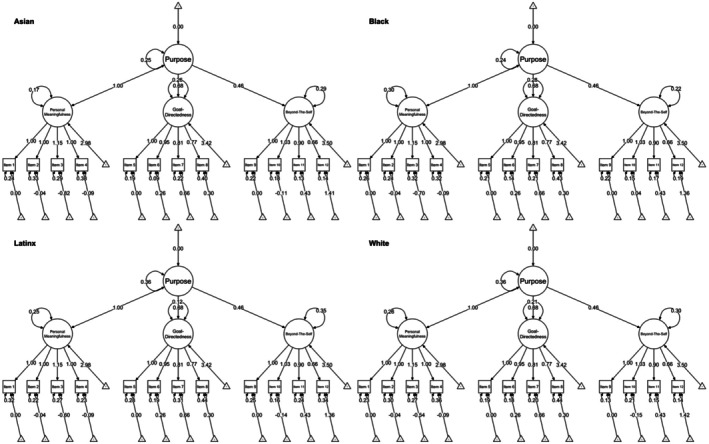
Second‐order model with partial scalar invariance (Model 9). Item 3, 10, and 12 intercepts freely estimated.

In summary, both CPS factor structures illustrated in Figure 1A,B demonstrated configural invariance, metric invariance, and partial scalar invariance across the four racial–ethnic groups in the present adolescent sample. The correlated three‐factor model also showed invariant inter‐factor covariances. The structure and parameter estimates of the final retained invariance models for the three‐factor model and the higher‐order model are presented in Figures 2 and 3, respectively.

### Potential racial–ethnic group differences in purpose and moderating effects

To examine potential racial–ethnic differences in CPS‐based purpose, we evaluated both group differences in latent means and potential moderation of structural associations. Building on the partial scalar‐invariant models (Model 3a and Model 9), latent mean differences were tested by comparing models with freely estimated latent means to models constraining latent means to equality across groups. Under the correlated three‐factor structure, constraining the latent means did not substantially worsen model fit (△*χ* ^2^ [9] = 5.87, *p* = .75). This pointed to no significant group differences across the three CPS dimensions. Under the second‐order structure, the comparison was conducted for the higher‐order latent mean (i.e., the purpose total score). Constraining the higher‐order mean likewise did not substantially change model fit (△*χ* ^2^ [3] =2.99, *p* = .40), suggesting no significant racial–ethnic differences in overall purpose.

Finally, multigroup SEM models were specified to examine whether racial–ethnic group membership moderated associations between CPS‐based purpose and related adjustment outcomes. Because this analysis involved examination of associations, measurement invariance of the outcome variables (depression, life satisfaction, and openness) was also evaluated, even though these variables were included at the observed level in the structural models. All outcome measures achieved at least metric invariance (see Table S1), supporting their inclusion in the multigroup structural analyses.

We tested potential moderation by comparing two sets of structural models: one in which the regression paths from CPS‐based purpose to the adjustment outcomes (i.e., depression, life satisfaction, and openness) were freely estimated across groups, and another in which these paths were constrained to be equal across groups (shown in Figures S2 and S3). Across both factor structures, constraining the structural paths did not significantly worsen model fit. In the correlated three‐factor model, in which the three CPS dimensions were specified as separate predictors, the constrained model did not differ from the freely estimated model significantly (Δ*χ* ^2^ [27] = 36.47, *p* = .11). The same pattern emerged in the second‐order model, in which the higher‐order purpose factor served as the predictor (Δ*χ* ^2^ [9] = 3.17, *p* = .96). All in all, this testing suggested no significant moderating effect of racial–ethnic group membership on the association between purpose and its adjustment outcomes.

Given this pattern of null results, the constrained models were retained, and their parameter estimates are shown in Table 3. Unstandardized estimates (Estimate b) are presented in the first column and are identical across groups. For the three‐factor structure, adjustment outcomes differed across purpose subscale. Specifically, only personal meaningfulness was found to be positively associated with life satisfaction and negatively associated with depression. In contrast, goal directedness and beyond‐the‐self orientation were only significantly associated with higher openness. When CPS‐based purpose was modeled as a single overarching construct, it was positively associated with life satisfaction and negatively associated with depression, but not significantly associated with openness.

**TABLE 3:** Parameter estimates for the multiple‐group SEM (constrained models).

Predictor	Outcome	Estimate b	SE	*p*	Std estimate β (Asian)	Std estimate β (black)	Std estimate β (Latinx)	Std estimate β (White)
Three‐factor structure								
Personal Meaningfulness	Depression	**−0.43**	**0.05**	**< .001**	**−0.47**	**−0.48**	**−0.59**	**−0.48**
	Life Satisfaction	**0.74**	**0.07**	**< .001**	**0.54**	**0.58**	**0.72**	**0.68**
	Openness	−0.02	0.06	.772	−0.02	−0.02	−0.02	−0.02
Goal Directedness	Depression	0.03	0.05	.625	0.03	0.03	0.02	0.03
	Life Satisfaction	−0.01	0.08	.949	0.00	0.00	0.00	0.00
	Openness	**0.16**	**0.07**	.**016**	**0.16**	**0.17**	**0.12**	**0.17**
Beyond‐The‐Self	Depression	0.00	0.06	.975	0.00	0.00	0.00	0.00
	Life Satisfaction	−0.14	0.08	.073	−0.09	−0.08	−0.10	−0.10
	Openness	**0.24**	**0.07**	.**001**	**0.22**	**0.19**	**0.22**	**0.22**
Second‐order structure								
CPS‐Purpose total	Depression	**−0.43**	**0.06**	**< .001**	**−0.47**	**−0.44**	**−0.59**	**−0.49**
	Life Satisfaction	**0.71**	**0.09**	**< .001**	**0.52**	**0.54**	**0.69**	**0.66**
	Openness	0.11	0.06	.062	0.11	0.12	0.14	0.14

*Note*: Because no evidence of moderation was found, the constrained models were retained and presented. The bolded rows indicate significant effects. Unstandardized estimates (Estimate b) are identical across groups, but standardized estimates (Std Estimate β) vary slightly due to group‐specific variances.

## DISCUSSION

Over the past several decades, research on positive youth development has increasingly recognized purpose as a vital developmental asset (Burrow & Hill, 2011; Scales et al., 2000). The Claremont Purpose Scale (CPS; Bronk et al., 2018), rooted in Damon et al. (2003) framework of youth purpose development, was designed to address the lack of purpose measures specifically targeting adolescents. However, previous validation studies on the English version scale have focused on emerging adults from predominantly white backgrounds, leaving uncertainty about the measure's utility for adolescents and its functioning across diverse racial–ethnic groups. The present study addressed these gaps by examining the measure's psychometric properties among adolescents and testing its measurement invariance across youth from diverse racial–ethnic backgrounds. We review each of our results in turn.

### Factor structure and convergent validity of the CPS

In the first part of our study, we tested two structural representations of the CPS. This was done to respond to Bronk et al. (2018) original intent to develop an instrument that could simultaneously function as an overarching factor or three related constructs, as well as to contribute to ongoing discussion regarding the CPS factor structure (see Veazey et al., 2023).

In our sample of U.S.‐based adolescents, both three‐factor correlated and higher‐order models demonstrated equivalently good model fit and strong item loadings. This supports Bronk et al. (2018) claims of the CPS operating as a flexible scale, in addition to providing support for the idea that individual CPS items function well as representations of their respective dimensions. However, examining the higher‐order structure provides additional nuance on the degree to which these dimensions integrated into an overarching purpose construct. Within the second‐order model, the loadings of personal meaningfulness (*λ* = .71) and goal directedness (*λ* = .64) were slightly lower than those reported by Bronk and colleagues (*λ*_emerging adult_ = .73 and *λ*_emerging adult_ = .90, respectively), though they remained within the range considered indicative of “good” construct representation (Comrey & Lee, 1992). A key difference emerged for the beyond‐the‐self dimension. In our adolescent sample, its loading on the higher‐order purpose factor was substantially lower (*λ* = .42) than that observed in Bronk and colleagues' emerging adult sample (*λ*_emerging adult_ = .68), indicating comparatively weaker integration with the overall purpose construct. In addition, within the correlated three‐factor model, associations involving beyond‐the‐self orientation were comparatively weaker than what was observed between personal meaningfulness and goal directedness alone.

One explanation for the weaker relevance of the beyond‐the‐self dimension in this sample lies in the fit of the scale's language with the developmental period. Many items within this dimension apply expansive and abstract phrasing (e.g., “broader world,” “a better world”), which may feel disconnected from adolescents' everyday experiences. Research on youth civic engagement suggests that while teenagers demonstrate competence in local engagement (e.g., community service), broader civic and sociopolitical involvement (e.g., electoral participation, political voice) typically increases with age and autonomy (Wray‐Lake et al., 2020). Practical constraints further limit adolescents' opportunities for large‐scale contribution, such as ineligibility to vote or being unable to drive independently to participate in community initiatives. Younger adolescents may instead express purpose through more concrete and proximal forms of contribution, such as supporting peers, engaging in school‐based leadership, or participating in local service (Fuligni, 2019). Because these tangible expressions are not directly reflected in current CPS items, the scale may be less sensitive to variation in beyond‐the‐self orientation in younger samples.

Two other explanations for the relatively low beyond‐the‐self orientation loading are rooted in an ongoing conversation about the role of beyond‐the‐self purpose content in adolescence. In short, there is tension between “having” and “sensing” approaches to purpose assessment (see Burrow et al., 2021; Damon, 2025). When purpose is defined in terms of “having” a beyond‐the‐self aim, prevalence estimates among youth tend to be low (e.g., ~25%; Moran, 2009). In contrast, “sensing” approaches typically find that youth possess moderate to high feelings of purposefulness (e.g., Burrow & Hill, 2011; Kiang, 2012). On one hand, our low loadings may indicate that the beyond‐the‐self orientation is an important—but perhaps not central or defining—aspect of youth purpose. Indeed, youth may hold different content aims that are all equally “purposeful” (see, for example, Hill et al., 2010). On the other hand, McKnight and Kashdan (2009) noted that purpose awareness (i.e., the extent to which one can consciously identify and articulate their purpose) varies in salience across individuals. This raises the possibility of a developmental arc for beyond‐the‐self manifestations of purpose, as well as developmental heterogeneity in that arc: adolescents may hold a meaningful and motivating *sense* of purpose early on, but it may take more time to fully express its *content*. If true, the lower relevance of the beyond‐the‐self dimension to other parts of purpose in our sample may reflect a conceptual opportunity to advance a united theory of beyond‐the‐self purpose development. Future work would be needed to confirm a developmentally grounded argument for beyond‐the‐self purpose, but our findings raise its possibility.

Although our findings raise important questions for purpose assessment literature and contribute to ongoing discussions regarding the CPS factor structure, it is worth reiterating that both CFA models demonstrated satisfactory performance with respect to global model fit. Consistent with the scale's original design, results supported the utility of the CPS for assessing purpose‐related constructs among adolescents as either three related dimensions or a single overarching factor. In exploratory analysis, however, an extended model based on the CPS's correlated three‐factor structure provided a significantly better representation of the data than the extended second‐order model. This pattern can be interpreted in both statistical and conceptual terms. Statistically, the second‐order extended model is more parsimonious, which may partly explain its poorer fit. Conceptually, however, the finding may suggest that modeling purpose as three related but distinct dimensions can provide additional information that is not fully captured by a single overarching construct—a feature of the scale that may be important to consider in future research that chooses to utilize it. That said, the current analyses cannot determine which explanation is more plausible; therefore, the superior fit of the correlated‐three factor model should be interpreted cautiously.

Finally, in terms of convergent validity, zero‐order correlations with theoretically relevant constructs occurred in expected directions: both the CPS total score and subscales scores were positively associated with openness, life satisfaction, and LET‐based purpose, and negatively associated with depression. Nevertheless, because the three CPS subscales were correlated with one another, these bivariate associations do not clarify whether each dimension has unique relations with these constructs after accounting for their shared variance as well as variance associated with certain covariates (e.g., age, gender). This issue was examined further in the subsequent analyses.

### Racial–ethnic measurement invariance of the CPS

Scholars have long proposed that marginalization may shape how adolescents develop and express purpose. For instance, Sumner et al. (2018) suggested that adolescents who face marginalization may be especially motivated to pursue civic purposes that address systemic issues affecting their communities. Similarly, research in cultural psychology indicates that people of color in the U.S. tend to endorse more collectivistic values (Coon & Kemmelmeier, 2001; Triandis, 1988), which could orient them toward beyond‐the‐self goals. Together, these perspectives raise the possibility that the beyond‐the‐self dimension of purpose varies in centrality across racial–ethnic groups.

However, our findings provided limited evidence for this perspective. We observed configural and metric invariance across groups, and partial scalar invariance after freeing a small number of item intercepts. Because partial invariance is common and widely accepted in practice (Putnick & Bornstein, 2016), we can conclude that the CPS operates in largely equivalent ways across adolescents from diverse racial–ethnic backgrounds. Establishing this level of MI provides a stronger basis for future cross‐group comparisons, as observed differences in CPS‐based purpose are less likely to reflect measurement artifacts.

### Exploring racial–ethnic group difference in purpose and moderating effects

The establishment of MI paved the way for our subsequent exploration of racial–ethnic group differences across the three dimensions of CPS‐based purpose, the total CPS‐based purpose score, and the links between CPS‐based purpose and adjustment outcomes. Our analyses revealed no significant mean‐level differences in purpose and no evidence of moderation by racial–ethnic group membership. These results suggest that, at least based on our sample, purpose may reflect a broadly shared developmental asset during adolescence rather than systematically favoring or disadvantaging any particular group.

Equally encouraging, the absence of moderation indicates that, regardless of their racial–ethnic background and marginalization experiences, levels of CPS‐based purpose were consistently associated with more adaptive adjustment outcomes. These findings underscore the widely protective role of purpose (see also Burrow & Hill, 2011; Goodwill, 2023; Malin et al., 2015; Ratner et al., 2023) and suggest that efforts to cultivate it may yield benefits for adolescents from a variety of backgrounds.

It is also worth noting that when testing predictors concurrently, SEM estimates suggested different associations between the CPS dimensions and adjustment outcomes among adolescents. Personal meaningfulness was associated with more favorable subjective well‐being, as indicated by higher life satisfaction and lower depression, whereas goal directedness and beyond‐the‐self orientation were more closely associated with openness, reflecting a greater willingness to think in the abstract and engage with new ideas and experiences. This pattern again resonates with Veazey et al. (2023) argument that CPS‐based purpose measurement may be more usefully conceptualized as three separate purpose‐related dimensions rather than as indicators of a single overarching purpose score, particularly when researchers are interested in predicting adjustment.

### Limitations and future directions

Despite providing new evidence of the utility of the CPS for adolescents from racially and ethnically diverse populations, several limitations should be considered when interpreting these results. First, the sample of our study was composed of adolescent participants preparing for a self‐driven learning program, so it is reasonable to wonder if these adolescents have stronger agency and purpose than their peers who did not apply to the GripTape program. Future research is warranted to validate the scale on adolescents from a broader range of contexts.

A second limitation is our exclusion of adolescents identifying as Native Hawaiian or Other Pacific Islander, Native American, multiracial, or an unspecified racial–ethnic background from the measurement invariance testing and subsequent analyses. We did this because of insufficient group sizes, but this decision made us unable to detect disparities that may exist between these participants and our four examined racial groups. Future research should strive to incorporate a more comprehensive representation of diverse racial–ethnic backgrounds, thereby enabling the establishment of invariance among all groups. Moreover, beyond focusing exclusively on racial–ethnic composition, the intersectionality of identity also warrants further investigation, as previous studies have demonstrated that factors such as gender and socioeconomic status play crucial roles in youth purpose development (Bronk et al., 2020; Gutowski et al., 2018; Sumner, 2020). Future research should examine how these social factors intersect and interact with race and ethnicity to shape adolescents' conceptualization and formation of CPS‐based purpose.

The third limitation concerns the age range of the current sample. Adolescence is marked by rapid developmental change, yet our sample was constrained to middle and late adolescence. Developmental research indicates that early adolescence differs meaningfully from later stages in terms of social and cognitive functioning (Steinberg, 2005), how they perceive themselves (Tamm et al., 2024), and in identity development processes (Meeus et al., 2012). Given that the CPS is intended “for use with adolescents,” it is therefore important to examine whether its psychometric properties are equally robust among younger adolescents and whether measurement comparability can be established across the full developmental period.

Fourth, although the present study focused on two theoretically grounded representations of the CPS, these two models do not fully resolve how the multidimensional structure of the CPS is best represented. Recent work in psychological measurement has contrasted hierarchical and bifactor models, which differ in their assumptions regarding the relative prominence of a general factor versus domain‐specific dimensions (e.g., Markon, 2019; Reise et al., 2010). Although bifactor structures have not been examined in prior CPS research, they could potentially offer an alternative framework for conceptualizing purpose by distinguishing shared variance among items from dimension‐specific variance. This may lend itself to insight into how each component of purpose uniquely contributes to well‐being (Hill et al., 2018). In addition to testing alternative factor structures, tools stemming from item response theory and graphical analyses may also provide complementary information about item‐level functioning and the pattern of associations among items in future investigation. Continuous examination of the psychometric properties of the CPS is therefore a warranted step toward advancing future research on youth purpose.

Lastly, although racial–ethnic measurement invariance of the CPS was examined, such tests address only statistical functioning rather than participants' subjective interpretations. In other words, MI tests do not establish whether adolescents from different backgrounds understand and interpret the items in the same way. Future research should complement the current study with qualitative approaches, such as cognitive interviews, to gather more direct evidence about how participants understand the construct (Balza et al., 2022). As noted by Bronk (2014), interviews are considered the “gold standard” in purpose research. Indeed, these methods may be better suited for determining whether items are interpreted similarly across groups.

## CONCLUSIONS

This study replicated and extended Bronk et al. (2018) initial validation of the CPS by examining the English version's psychometric performance in a racially and ethnically diverse U.S. adolescent sample. Specifically, we demonstrated that two mathematically equivalent factor structures, each reflecting a distinct theoretical representation of the CPS, showed good overall fit and zero‐order associations with adjustment outcomes. These findings suggest that researchers may use the scale either as separate subscales or as a general factor, depending on their goals.

However, two important caveats to these findings are still worth noting. First, our exploratory CFA extension suggested that using the CPS as three correlated factors may be more informative for adolescents. This is an especially important point given our subsequent testing that showed adjustment outcomes may vary by subscale domain. For example, personal meaningfulness seemed to carry the “weight” of purpose's association with youth well‐being. Second, the weaker relevance of the beyond‐the‐self dimension to the other dimensions and the higher‐order purpose construct opens space for conversation about how youth purpose should be defined and measured. This latter nuance may be particularly relevant for conversations about the centrality and developmental arc of beyond‐the‐self concerns during adolescence.

Finally, by establishing measurement invariance and finding no evidence of racial–ethnic differences in either CPS‐based purpose levels or its associations with adjustment outcomes, this study advances a more inclusive understanding of adolescent purpose development. It responds to concerns about race‐neutral assumptions in psychological science (Dupree & Kraus, 2022) by empirically examining whether purpose operates similarly across racial–ethnic groups, rather than presuming that findings derived from predominantly white samples are universally generalizable (e.g., Syed et al., 2018). In doing so, this work paves the way for a more inclusive and confident science of youth purpose.

## AUTHOR CONTRIBUTIONS

**Melody Estevez:** Project administration; resources. **Hou Xie:** Conceptualization; data curation; investigation; methodology; formal analysis; visualization; writing – original draft; writing – review and editing. **Anthony L. Burrow:** Conceptualization; funding acquisition; writing – review and editing; supervision. **Kaylin Ratner:** Conceptualization; investigation; methodology; writing – review and editing.

## FUNDING INFORMATION

This project has been made possible in part by a grant from the Chan Zuckerberg Initiative (No. 136823), an advised fund of Silicon Valley Community Foundation. Any findings, opinions, or recommendations expressed in this paper are those of the authors and do not necessarily reflect the views of the funder. The funder played no role in study design, analysis of data, interpretation of results, or writing of the final report.

## CONFLICT OF INTEREST STATEMENT

Readers should be advised that one co‐author (M.E.) is a paid employee of GripTape. Any findings, opinions, or recommendations expressed in this paper are those of the authors and do not necessarily reflect the views of the GripTape organization.

## ETHICS STATEMENT

All procedures were approved by Cornell University (supervising institution) Institutional Review Board. The protocol number was IRB#2011009919 (approved in August 2020).

## CONSENT

Consent was obtained from all participants or their guardians (if participants were under 18) prior to survey administration.

## Supporting information


**Table S1:** Model fit indices and changes in fit across measurement invariance models of adjustment outcomes.
**Figure S1:** Extended correlated three‐factor and second‐order CFS models with external criterion variables.
**Figure S2:** Conceptual model of the multiple‐group SEM (Three‐factor structure).
**Figure S3:** Conceptual model of the multiple‐group SEM (Second‐order structure).

## Data Availability

Data and materials that support the findings of this study are available from the corresponding author upon reasonable request and in consultation on a case‐by‐case basis with our practitioner partners.

## Footnotes

^1^
We thank a reviewer for their suggestion during the review process, as well as for prompting us to confirm that the two extended CFA models were indeed nested.
