# Supplementary material for: Establishing validity and measurement invariance of the Claremont Purpose Scale among adolescents from diverse racial–ethnic backgrounds
Source: J Res Adolesc. 2026 Jun 8;36(2):e70215. doi: 10.1111/jora.70215 (PMC13247353; doi:10.1111/jora.70215)
Supplement: Supplementary file 1 — Table S1: Model fit indices and changes in fit across measurement invariance models of adjustment outcomes. Figure S1: Extended correlated three‐factor and second‐order CFS models with external criterion variables. Figure S2: Conceptual model of the multiple‐group SEM (Three‐factor structure). Figure S3: Conceptual model of the multiple‐group SEM (Second‐order structure). [file JORA-36-0-s001.docx]

**Table S1**

*Model Fit Indices and Changes in Fit Across Measurement Invariance Models of Adjustment Outcomes*

|  | *χ2(df)* | CFI | TLI | RMSEA | MNCI | △*χ2(*△*df)* | △CFI | △MNCI | △RMSEA | RMSEA_D_ |
| --- | --- | --- | --- | --- | --- | --- | --- | --- | --- | --- |
| Depression |  |  |  |  |  |  |  |  |  |  |
| Mode 1 Configural invariance^†^ | 1077.94 (660) | 0.90 | 0.89 | 0.08 | 0.58 |  |  |  |  |  |
| Model 2 Metric invariance^†^ | 1123.51 (711) | 0.90 | 0.90 | 0.07 | 0.59 |  |  |  |  |  |
| Model 1 – Model 2 |  |  |  |  |  | 42.95(51) | 0.001 | 0.004 | -0.005 | 0.000 |
|  |  |  |  |  |  |  |  |  |  |  |
| Life Satisfaction |  |  |  |  |  |  |  |  |  |  |
| Mode 1 Configural invariance | 24.55 (20) | 1.00 | 0.99 | 0.05 | 0.99 |  |  |  |  |  |
| Model 2 Metric invariance | 34.46 (32) | 1.00 | 1.00 | 0.03 | 0.99 |  |  |  |  |  |
| Model 1 – Model 2 |  |  |  |  |  | 9.40(12) | 0.002 | 0.002 | -0.020 | 0.000 |
|  |  |  |  |  |  |  |  |  |  |  |
| Openness |  |  |  |  |  |  |  |  |  |  |
| Mode 1 Configural invariance | 3.45 (4) | 1.00 | 1.01 | 0.00 | 1.00 |  |  |  |  |  |
| Model 2 Metric invariance | 10.30(13) | 1.00 | 1.02 | 0.03 | 1.00 |  |  |  |  |  |
| Model 1 – Model 2 |  |  |  |  |  | 6.62(9) | 0.000 | 0.002 | 0.000 | 0.000 |

*Note.* The depression models were specified based on the three-factor structure identified by Faulstich et al. (1986). ^†^Model allowing correlated residuals between items 17 and 18, as well as item 14 and 15.

**Figure S1**

*Extended correlated* *three-factor and second-order CFS models with external criterion variables*

**
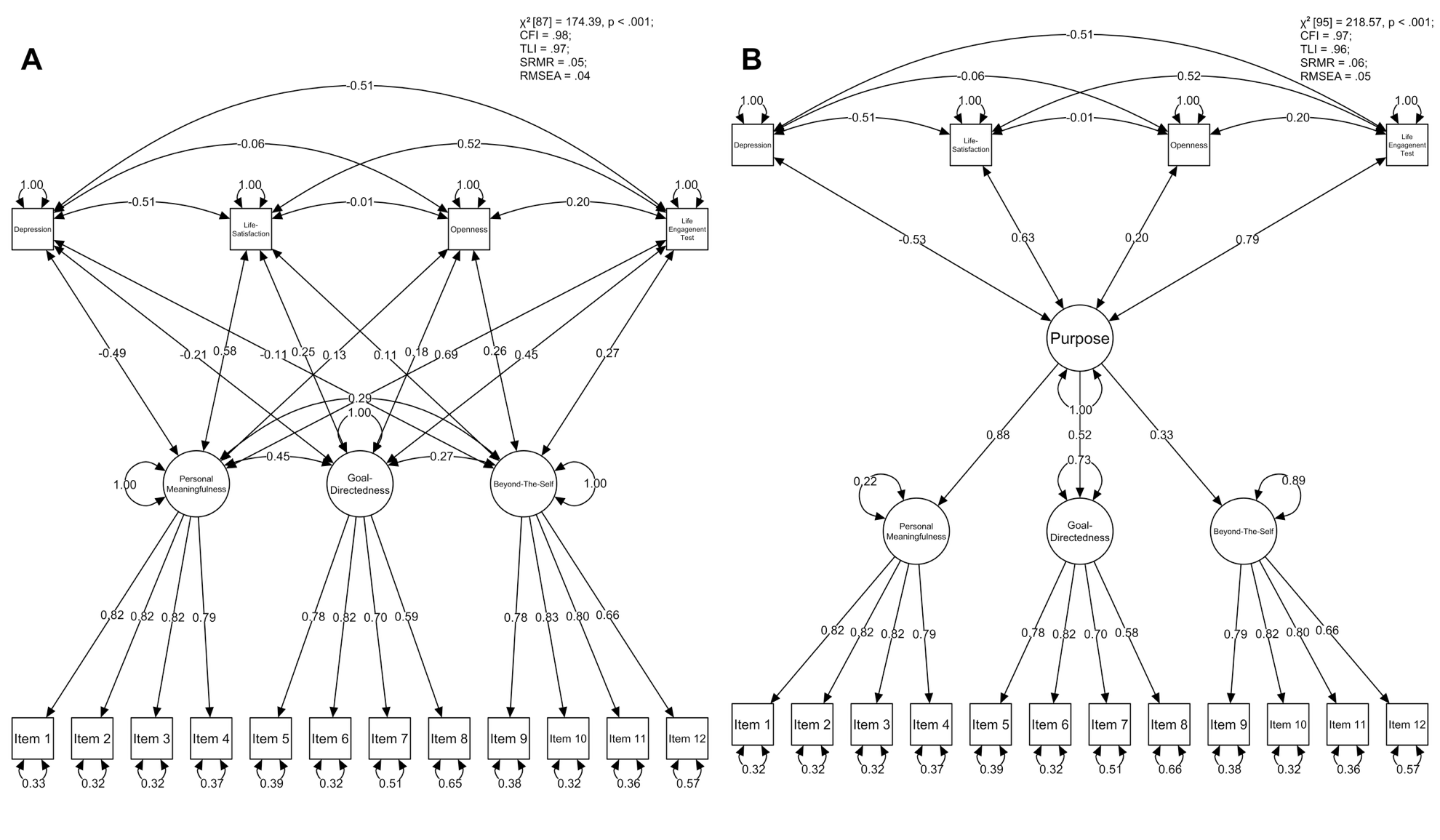
**

**Figure S2**

***Conceptual Model of the Multiple-Group SEM (Three-Factor Structure)***

*
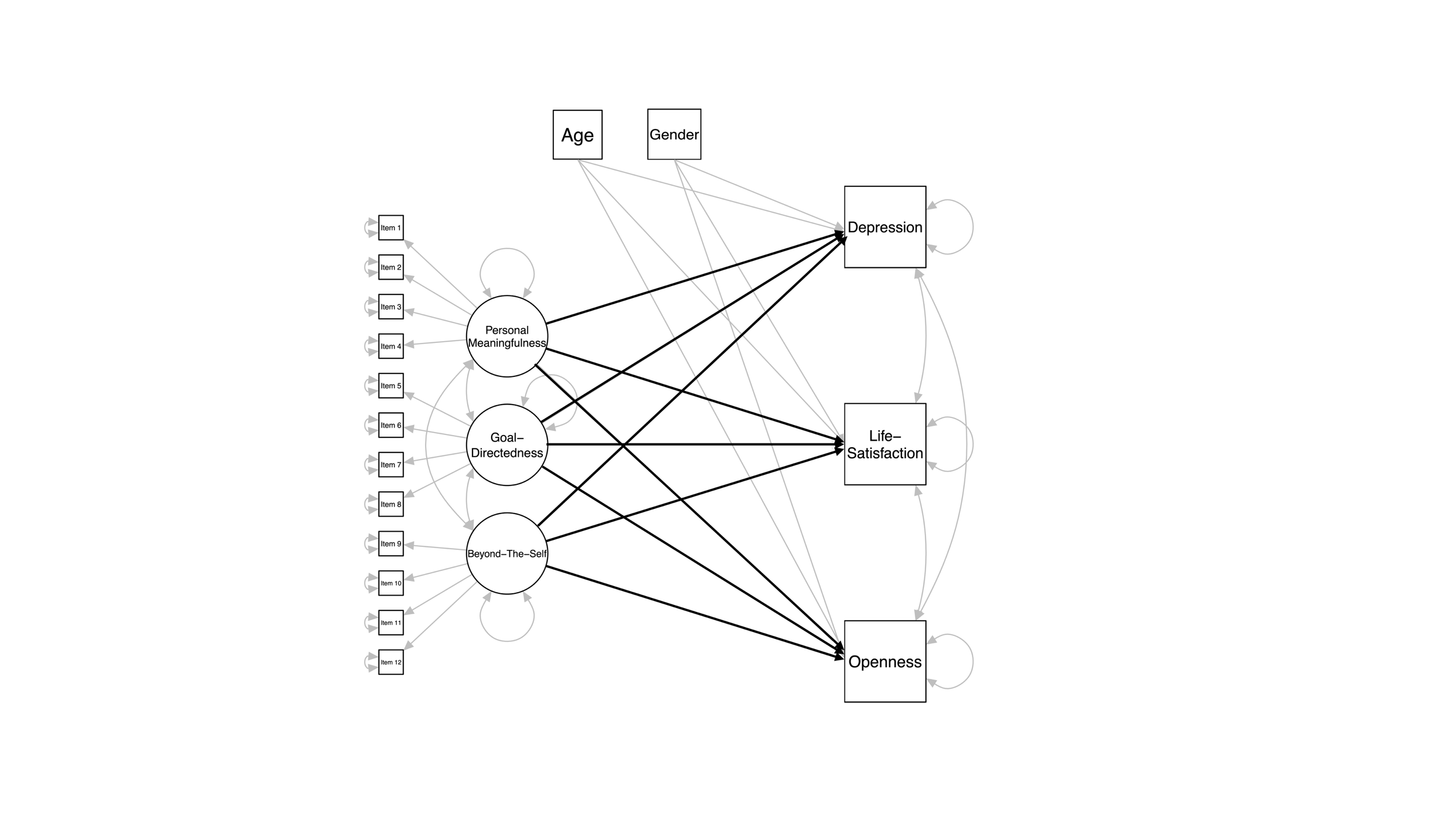
*

*Note.* All estimated paths are shown in gray. Paths of focal interest are highlighted in black. In the multiple-group SEM analyses, these focal paths were constrained to be equal across groups in the constrained model and freely estimated in the unconstrained model.

**Figure S3**

***Conceptual Model of the Multiple-Group SEM (Second-Order Structure*)**

**
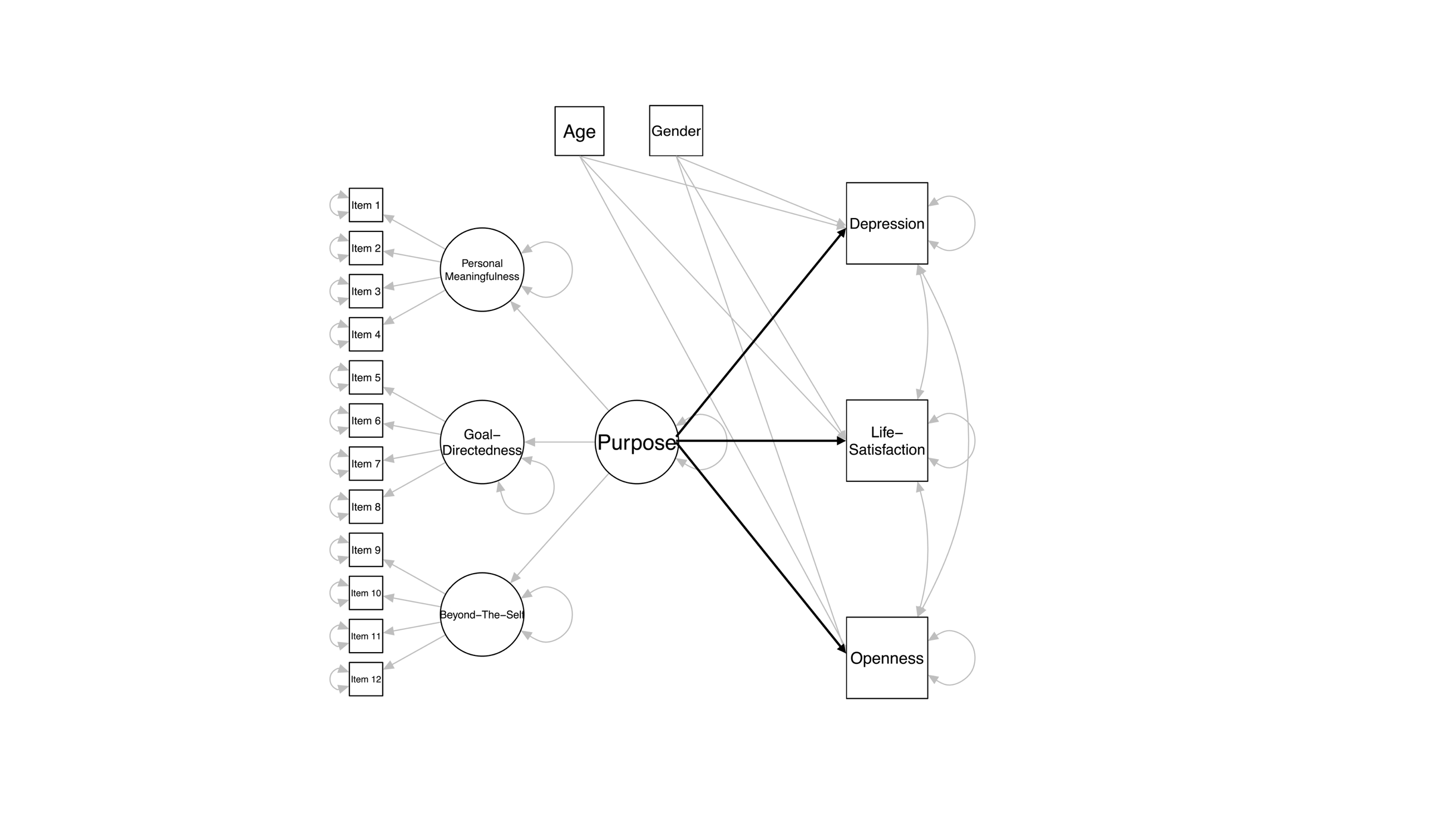
**

*Note.* All estimated paths are shown in gray. Paths of focal interest are highlighted in black. In the multiple-group SEM analyses, these focal paths were constrained to be equal across groups in the constrained model and freely estimated in the unconstrained model.
